# The Ethics of Ozempic and Wegovy

**DOI:** 10.1136/jme-2024-110374

**Published:** 2025-01-23

**Authors:** Nanette Ryan, Julian Savulescu

**Affiliations:** 1 National University of Singapore Centre for Biomedical Ethics, Singapore; 2 Centre for Biomedical Ethics, Yong Loo Lin School of Medicine, National University of Singapore, Singapore; 3 Uehiro Oxford Institute, University of Oxford, Oxford, UK

**Keywords:** Ethics, Ozempic, Semaglutide, Weight loss

## Abstract

Semaglutide, sold under the brand names of Ozempic, Rybelsus and Wegovy, is one of the most popular drugs on the market. Manufactured by Novo Nordisk, semaglutide is the newest in a family of glucagon-like peptide-1 receptor agonists used most commonly to treat type II diabetes. To date, the results of semaglutide for the treatment of type II diabetes have been overwhelmingly positive. It is for the drug’s effects on appetite suppression and weight loss, however, that have led its surge in popularity, with many hailing semaglutide as the new ‘miracle drug for weight loss’. Despite its popularity, both the governmental and popular reception to the drug has largely been mixed. In this paper, we address a range of ethical concerns and argue that while many are legitimate, they do not provide conclusive reason not to prescribe semaglutide for weight loss.

## Introduction

 Semaglutide, sold under the brand names of Ozempic, Rybelsus and Wegovy, is one of the most popular drugs on the market. Manufactured by Novo Nordisk, semaglutide is the newest in a family of glucagon-like peptide-1 (GLP-1) receptor agonists used most commonly to treat type II diabetes. To date, the results of semaglutide for the treatment of type II diabetes have been overwhelmingly positive. It is for the drug’s effects on appetite suppression and weight loss, however, that have led its surge in popularity, with many hailing semaglutide as the new ‘miracle drug for weight loss’.^1^

Despite its popularity, both the governmental and popular reception to the drug have largely been mixed. Some commentators are concerned that semaglutide will be used as an ‘easy way out’ at the individual and governmental level, ignoring the underlying causes of the health issue. Professor Giles Yeo, a geneticist at the University of Cambridge, for example, comments, ‘I do fear…that actually not only our government, but many governments and policymakers, may very well use [these drugs] as a cop-out not to make the hard policy decisions. And that is a real issue’.^2^ Yeo adds ‘[weight loss drugs] make you feel fuller: you feel fuller, you eat less…But what they don’t do is improve your diet’.^2^ Some commentators are concerned that the surge in popularity of semaglutide will leave those who need it most for therapeutic purposes worse off. As research professor Flora Oswald describes ‘…the surge of interest in the off-label fat loss effects of Ozempic and similar drugs has resulted in a worldwide shortage of these drugs, rendering diabetes patients unable to access life-sustaining medication’.^3^ And others are concerned that the widespread use of semaglutide for weight loss will reinforce an already harmful fat-phobic rhetoric. Senior lecturer in nutrition, dietetics and food innovation, Dr. Emma Beckett, writes ‘drugs like Ozempic won’t “cure” obesity but they may make us more fat-phobic’.^4^

In this paper, we address these ethical concerns and argue that while many are legitimate, they do not provide conclusive reason not to prescribe semaglutide for weight loss. Moreover, we argue that there are good *prima facie* reasons to prescribe semaglutide for weight loss in so far as doing so has other therapeutic weight-related benefits, beyond those for the treatment of type II diabetes. Prescribing semaglutide for weight loss, we argue, also has the potential to promote persons’ autonomy and can minimise the harms of state-mandated shortages. That said, we also contend that when prescribing semaglutide, priority should be given to patients when it is clear that their health needs for the medication outweigh alternative uses. This may mean that treatment for type II diabetes is prioritised over treatment for obesity. However, we contend that it will not necessitate prioritising treatment for type II diabetes given the benefits semaglutide can provide for obesity health-related issues, and the complexity of individual health profiles.

We begin discussing the function and efficacy of semaglutide for treatment of type II diabetes and weight loss. We then consider the positive case for the prescription of semaglutide for weight loss, and end by evaluating four common objections.

The assessment criteria we employ in our analysis focus on the benefits, risks and costs (including financial, psychological and physical health-related costs) of semaglutide use, as well as the values of social equality, fairness and autonomy. We take these to be fundamental ethical parameters for analysis. We acknowledge, however, that other assessment criteria may be used. Whether alternative criteria would provide the same ethical conclusions is beyond the scope of this paper. However, we suspect that any widely agreed-upon alternatives would arrive at similar ethical conclusions.

## The science + potential

In 2017, Ozempic was first approved for use in the USA to treat type II diabetes. In 2021, Wegovy was also approved for the US market but as a weight loss drug. According to Novo Nordisk, Ozempic is ‘an adjunct to diet and exercise to improve glycaemic control in adults with type 2 diabetes mellitus’.^5^ Wegovy is ‘for adults with obesity (body mass index (BMI) ≥ 30) or overweight (excess weight) (BMI ≥ 27) who also have weight-related medical problems, to help them lose weight and keep it off’.^6^ Ozempic and Wegovy are taken via a single-dose weekly injection (0.5 mg to 2 mg and 2.4 mg per dose, respectively).^[^^i^^]^

Semaglutide functions to treat type II diabetes in a number of ways. First, it assists in the control of blood glucose levels by mimicking the GLP-1 hormone which helps the production of insulin when blood glucose levels are elevated—insulin helps to move the glucose out of the bloodstream (extracellular) and into cells (intracellular), and when it does, so blood glucose levels decrease. Semaglutide also slows gastric emptying and suppresses appetite by interacting with parts of the brain that signal hunger and satiation. This functions to treat diabetes as it encourages patients to consume fewer calories to be converted to glucose. Suppression of appetite also encourages weight loss. While the reasons people develop type II diabetes are not entirely clear, being overweight does play an important role in a person’s risk of developing type II diabetes and their ability to manage it.^7^ This is because when people have a high BMI, their body sometimes requires ‘as much as two to three times more insulin’ than someone with a ‘healthy’ BMI.^8^ These levels of insulin are typically more insulin than a person’s pancreas is able to produce, and so having a high BMI can contribute to a person’s ability to maintain healthy glucose levels.

As a drug to treat type II diabetes and weight loss, semaglutide has seen great success. With respect to type II diabetes, in one clinical trial (the Sustain 7 Study), patients with type II diabetes taking Ozempic showed a reduction in haemoglobin A1c—a measure of average blood sugar—of up to 1.8% over 40 weeks.^9^ A person with 5.7% is in the normal range, 5.7%–6.4% is indicative of prediabetes, and 6.5% or higher indicates diabetes.^10^ The haemoglobin A1c change seen in the Sustain 7 Study then can move someone from the range of having diabetes, to a normal 5.7%. In addition to these benefits, studies have shown evidence of a reduced risk of heart attack, stroke and kidney failure in patients with type II diabetes.^11 12^

For people taking semaglutide for weight loss, the results so far have also been significant. In one 2-year randomised control trial (Semaglutide Treatment Effect in People 5 (STEP 5)) of 304 participants, participants were shown to have lost, on average, −15.2% of their body weight when taking Wegovy, compared with −2.6% taking the placebo. The participants were overweight adults (with BMI ≥ 27 kg m^–2^) or adults with obesity (with BMI ≥ 30 kg m^–2^), with at least one weight-related comorbidity, without diabetes.^13^

In addition to these outcomes, one time per week use of 2.4 mg of semaglutide has also been shown to be beneficial for the cardiovascular health of obese people without diabetes. In one double-blind, randomised, placebo-controlled study (the SELECT trial), ‘17 604 adults aged 45 years or older with overweight or obesity and established cardiovascular disease (CVD) with no prior history of diabetes’ were given 2.4 mg of semaglutide for up to 5 years.^14^ Results showed a 20% improvement in overall risks of major cardiac events including heart attack, stroke and cardiovascular death.^15^ Participants also showed a reduced risk of 28% with respect to a reduction of non-fatal myocardial infarction, 15% of cardiovascular death and 7% of non-fatal stroke.^15^ According to Yale cardiologist Harlan Krumholz, the result of ‘the SELECT trial is a major breakthrough’ because it demonstrates that the use of semaglutide is an effective treatment for cardiovascular disease, independently of the presence of type II diabetes.^16^ Importantly, ‘mechanisms of cardiovascular risk reduction with semaglutide may include those related to physiological benefits from the reduction of excess abnormal body fat and actions of semaglutide other than weight loss’.^14^ The reductions in cardiovascular risks observed in other trials with similar weight-loss reductions, as Lincoff *et al.* explain, were consistent with the findings here.^14^ They also claim that ‘more rapid treatment-induced physiological changes beyond the magnitude of body-weight loss may have mediated at least part of the cardiovascular benefit’.^14^ However, they acknowledge that this link is only speculative.

## Personal experiences

To date, there are no formal studies, comprehensive or otherwise, on the personal experiences of semaglutide beyond side effects documented in the efficacy studies. There are, however, many anecdotal accounts from private reports to celebrity endorsements and otherwise. Former politician and Prime Minister of the UK Boris Johnson, for example, reports that when taking Ozempic ‘I started to dread the injections, because they were making me feel ill’.^17^ In favour of the drug, American host and producer Oprah Winfrey reports that ‘instead of gaining eight pounds like I did last year, I gained half a pound…It quiets the food noise’.^18^

With respect to private reports, on drugs.com, a free drug-information service, 54% of reviewers of 2023 report having a positive experience with the drug, and 24% report a negative experience.^19^ 1160 reviewers were using the drug for type II diabetes, 646 for weight loss and 208 for cardiovascular risk reduction. One positive reviewer reports that ‘everyone gets the same side effects: sulfur burps, intense pain once a week—before #2, heartburn, diarrhoea (from fatty or dairy foods) and constipation…maybe forgetfulness, shivers, etc. However, for me, benefits outweigh the side effects!…’ (Benef, July 2023). Another reports that ‘first 2 weeks I had some mild nausea, nothing terrible and some mild dizziness. The past 2 weeks, I have had no symptoms…my sweet tooth cravings are pretty much gone. Really happy so far…’ (Dana, September 2020).^19^ One negative response reports that ‘the side effects of my gastrointestinal symptoms have made this drug intolerable to me. While my A1C is now down to 6.1 from 7, at my age, 7 is still considered an acceptable result. So in my estimation, it has not been worth the severe cramps, diarrhoea, headaches, blurred vision and dizziness I have experienced’ (Margie, 3 July 2023).^19^ Another reports that ‘after 1 year of taking this, I began suffering from severe abdominal pain…I had to have my gallbladder removed. I gained the 50 pounds back that I had lost the previous year. Although it was effective, it was not worth the side effects’ (Helene, 19 June 2023).^19^ These reports are reflective of many more. To get a clear picture, however, of the personal experiences of patients, more research is needed.

## Governmental reception

Governmental reception of semaglutide has been mixed. In 2017, Ozempic was first approved for use in the USA to treat type II diabetes. In 2021, Wegovy was approved for the US market. Since their approval in the USA, many other countries including Canada, Japan, Australia, the UK and Singapore have followed suit approving one or both drugs for use. While both Ozempic and Wegovy are widely approved for use, many nations—including Canada, the UK, New Zealand and Germany—will only subsidise Ozempic.

According to Canadian regulators, Canada will not fund Wegovy as it concluded that, even though patients treated with Wegovy lost body weight, there is ‘no evidence to show that this weight loss translates to improvement in weight-related comorbidities (eg, cardiovascular complications, osteoarthritis [the most common form of arthritis], and sleep apnoea)’.^20^ In a similar vein, Emily Field, head of European Pharmaceuticals Equity, explained that the wider European market ‘[public health services] don’t want to pay for [Wegovy] if it won’t tackle underlying health conditions’.^21^ The suggestion here is that if semaglutide only has effects for weight loss in the absence of health benefits—merely a ‘vanity drug’ in Field’s words—then public health services would not cover it. However, the SELECT trial provides evidence that this is not the case.^21^

Germany also has announced that it will not publicly fund Wegovy as part of its wider ban on funding drugs aimed at dieting, appetite control and weight loss. According to a spokesperson for Germany’s health ministry ‘since such uses [of weight loss medications] are considered a matter of individual responsibility and personal lifestyle, these medications are not statutorily financed by the solidarity-based community of those insured’.^21^ Denmark has also announced that they will not fund Wegovy on the grounds that ‘its cost was incommensurate with its therapeutic value’.^21^

## Public reception

Public reception of semaglutide has also been mixed. On the one hand, there has been a surge of popularity of semaglutide, primarily for its efficacy as a weight loss drug. At the same time, however, those who take it for weight loss are often shamed for taking ‘the easy way out’. Rosenfield, author of ‘Why Ozempic is Cheating’, writes ‘whatever one’s reasons for losing weight, the common wisdom is that it’s not supposed to be easy, physically or mentally’.^22^ Popular reporting on the drug also describes people as ‘admitting’ use: ‘Rebel Wilson has admitted she took Ozempic to help her lose weight’, ‘dozens of A-listers have admitted using semaglutide…’^23 24^ Other popular figures have shown disdain for being associated with the drug on account of having their weight loss efforts undermined.^25^ This same treatment has not been extended to those who use semaglutide to treat type II diabetes or for cardiovascular health—‘health conditions’ that are presumably understood as a legitimate use of healthcare resources.

## Four reasons why semaglutide should be prescribed for weight loss

While the reception of Ozempic and Wegovy have been mixed, there are four important reasons why semaglutide should be prescribed for weight loss. As we will explain below, semaglutide is an effective form of therapy for those who need it, it provides a safer treatment option relative to other more aggressive therapies, its prescription can minimise scarcity-related harms, and it can be an effective tool to promote and respect autonomy.

### Improving health outcomes: semaglutide as therapy

Obesity is a complex condition caused by a range of factors including genetics, lifestyle and socioeconomics. Despite its complexity, obesity has a long history of being equated to poor health.^26^ New research, however, has shown that obesity simpliciter is not an accurate gauge of poor health. As researcher and writer, Couzin-Frankel explains there is evidence to suggest that the correlation between poor health outcomes and obesity correlates not to obesity in general, but to a particular kind of fat, namely, visceral adipose tissue (VAT).^27^ VAT rather than subcutaneous fat ‘generates inflammatory molecules, and imaging studies have shown it’s associated with fat build-up in the liver, pancreas, and muscle’.^27^

There is good evidence that semaglutide is therapeutically beneficial for persons in terms of a reduction in VAT. In one 68-week randomised control trial study (STEP 1) of 140 participants analysing the effects of semaglutide on the body composition of obese adults, participants displayed a −15.0% change in body weight at the end of the trial with semaglutide, compared with a −3.6% change with the placebo. Those taking semaglutide lost, on average, −19.3% of total fat mass and −27.4% of visceral fat mass.^28^ Insofar as VAT is problematic for health then, semaglutide could be an effective therapeutic treatment. It is important to note that it is unclear whether semaglutide has a direct impact on VAT reduction more than, say, other weight loss therapies that achieve the same total weight loss results. What is clear, however, is that semaglutide encourages total weight loss, which includes significant reductions in VAT. It also reduces lean mass, making it unsuitable for some patients, especially those for whom lean mass reductions could be harmful.

In addition to these results, as discussed earlier, patients in the SHAPES trial taking semaglutide showed significant improvement in overall risks of major cardiac events including heart attack, stroke and cardiovascular death, even for those persons without type II diabetes. For those persons at risk of experiencing health related problems with high levels of visceral fat, or cardiovascular issues, semaglutide could be prescribed as a very effective and potentially life-saving therapy.^7^

### Semaglutide as less risky therapy

Restricting access to semaglutide may also place persons unnecessarily at a higher risk of harm by limiting therapeutic options to more aggressive treatments or less effective options.

For some persons presenting with higher levels of VAT and cardiovascular issues, there is good evidence that traditional lifestyle changes (ie, modifications in diet and exercise) can improve health.^29^ For others, however, traditional lifestyle changes will be insufficiently effective. It is, as we described earlier, a complex condition caused by factors including genetics, lifestyle, socioeconomics, as well as an individual’s own metabolic rate, and physical capacity. As such, for some people, medical intervention may be required.

One of the most well-known and effective kinds of medicalised treatments for fat loss is bariatric surgery. ‘Bariatric surgery’ refers to a range of procedures including gastric band surgery, gastric bypass, sleeve gastrectomy and duodenal switch procedures.^30^ They aim to modify a person’s digestive system to reduce the number of calories that they consume to reduce body fat. One study published in 2024 examining the effects of various forms of bariatric surgery on 58 patients showed decreases in VAT of almost 60% in with Roux-en-Y gastric bypass (RYGB) and laparoscopic sleeve gastrectomy (LSG) over the course of the nearly 3-year study (Henry *et al*). Previous work, Henry *et al.* claim, ‘has demonstrated a reduction in VAT of 65%–77% 12 months post RYGB or LSG’.^31^ These studies then indicate a visceral fat loss of nearly double the results of semaglutide (2.4 mg) with a reduction of −27.4% of visceral fat mass.

Bariatric surgery, like semaglutide, comes with risks and side effects. Common side effects include nutritional deficiency, which can lead to conditions such as anaemia, osteoporosis, neuropathy, calcium oxalate urolithiasis and infertility.^30 32 33^ Other side effects include bleeding, infection, blood clots, hernias, bowel adhesions and obstruction, anastomotic leaks, dumping syndrome—that is, ‘when your stomach dumps food too fast into your small intestine’, resulting in symptoms including ‘nausea, diarrhoea, abdominal cramping and hypoglycaemia’—bile reflux, stomach ulcers and gallstones.^32^ In rare cases, surgery-related issues also lead to death.^34^

Common side effects of semaglutide include gastrointestinal issues, such as nausea, vomiting, diarrhoea, abdominal pain, bloating and constipation.^15^ In two placebo-controlled trials of participants with type II diabetes, of 261 participants taking 1 mg of semaglutide 20.5% experienced nausea, 9.2% experienced vomiting and 8.8% experienced diarrhoea, compared with 6.1%, 2.3% and 1.9% of 262 participants taking the placebo respectively. Other common side effects include headaches, weakness when exercising and what has been commonly termed ‘Ozempic face’.^35^ ‘Ozempic face’ refers to the sagging and ageing of facial skin caused by rapid weight loss that semaglutide encourages. According to Dr. Silvana Obici, Chief of Endocrinology and Metabolism Division at Stony Brook Medicine, ‘the loss of fat tissue from the face is very common with any weight loss, especially when is significant (15 or>20% of body weight)…thus people who lose weight may look more wrinkled and aged’.^36^

More serious but rare side effects of taking semaglutide include gastroparesis (stomach paralyses) and anhedonia (depression). Potential long-term side effects also include a heightened risk of thyroid C-cell tumours (adenomas and carcinomas), pancreatitis, kidney failure, gallbladder disease, and diabetic retinopathy.^37 38^

The kinds of adverse effects across treatments are not insignificant—especially, although rare, the risk of developing thyroid cancer—making neither appear as obviously the less risky choice. An important difference, however, is the rate of incidence. As Klair *et al.* explain in their ‘systematic review of clinical trials, including the STEP (Semaglutide Treatment Effect in People) trials, sustain trials, pioneer trials, and the STAMPEDE (Surgical Treatment and Medications Potentially Eradicate Diabetes Efficiently) trial’, bariatric surgery was found to have both a higher risk of complications and a higher incidence rate of adverse effects.^32^ Thus, they describe bariatric surgery as having the comparatively ‘higher risk profile’.

Bariatric surgery has been practiced for significantly longer than semaglutide as a therapy. The first bariatric surgery for weight loss, for example, occurred in the USA in the 1950s.^39^ The STEP 1 trial of semaglutide, conducted by Novo Nordisk, began in 2018. As such, we have, in some ways, a clearer picture of the long-term side effect profile of bariatric surgery than semaglutide. Importantly, however, there have also been no formal studies on the long-term efficacy and side effects of bariatric surgery. Additionally, ‘a lack of standardised reporting of complications or adverse events after bariatric procedures makes it difficult to clearly establish overall and specific rates of postsurgical problems’.^34^ So, while the risk assessment currently falls in favour of semaglutide, the accuracy of the risk profiles of both treatments are limited and may change over time with standardised study and analysis.^[ii]^

In addition to these considerations is the fact that semaglutide treatment can be easily discontinued, whereas it is much harder to reverse bariatric surgery (and sometimes impossible as in the case of gastric sleeve surgery). As such, if the side effects of either treatment prove to be intolerable, a benefit of semaglutide is that it may be stopped relatively quickly by ceasing treatment.

Not all patients who would benefit from medicalised fat loss will require the high VAT loss percentage that bariatric surgeries would provide to improve their health. However, bariatric surgery is, unlike the use of semaglutide, often prescribed and subsidised, provided that patients meet the assigned criteria. In Australia, for example, ‘Bariatric surgeries in the public sector are publicly funded’, and to qualify, a patient must have a (BMI) ‘greater than or equal to 35 kg/m² with at least one obesity-related comorbidity, and the patient should have had at least 6 months of medical treatment that did not work’.^40^ If we take Klair *et al.*’s analysis as accurate thus far, this means that those who would benefit from medicalised fat loss may, due to lack of availability, take on higher-risk procedures than would otherwise be necessary should semaglutide be made more available.

Another alternative to bariatric surgery are alternative GLP-1s, such as Saxenda (liraglutide). Saxenda is also a GLP-1 receptor agonist manufactured by Novo Nordisk. It is administered via a daily injection containing a starting dose of 0.6 mg of liraglutide. Saxenda has a similar risk profile to semaglutide. In one randomised clinical trial (STEP 8) comparing the effects of taking semaglutide weekly to Saxenda (liraglutide) on body weight in adults with overweight or obesity without diabetes, results showed that among the 338 participants, the most common adverse events for both medications were gastrointestinal disorders, with 84.1% of participants taking semaglutide reporting events, 82.7% for liraglutide and 55.3% for the placebo.^41^ Gallbladder-related disorders were also reported, with 0.8% for semaglutide, 3.1% for liraglutide and 1.2% for the placebo. Only one participant reported subclinical pancreatitis; they were from the liraglutide group and did not require treatment. In addition to the risk of pancreatitis and gallbladder-related disorders, both medications carry a risk of thyroid tumours.^42^ Saxenda and semaglutide also both have other mild side effects such as dizziness and injection site reactions and more serious effects that include increased heart rate, hypoglycaemia (low blood sugar) and dehydration.^43^

Saxenda is cheaper than Ozempic and Wegovy. In Singapore, for example, a low dose of Saxenda can cost as low as $2390 annually, whereas Ozempic can cost between $3466 and $4333.33 annually.^44^ It is also, however, significantly less effective. The results of the STEP 8 trial, for example, showed participants means weight change over 68 weeks at −15.8% with semaglutide, −6.4% with liraglutide and −1.9% with the placebo.^41^ In another study, a 3-year randomised, placebo-controlled study of 235 participants, the ‘mean change in VAT over a median of 36.2 weeks was −12.49% with liraglutide compared with −1.63% with placebo’.^45^ The total mean BMI change was −6.53% with liraglutide and −1.08% with placebo. In the STEP 1 trial, recall that participants taking semaglutide lost an average of −27.4% of visceral fat mass over 68 weeks, with a change in BMI of −15.0% at the end of the trial, compared with a −3.6% change with the placebo. While these trials are of different lengths, if we take STEP 8 as indicative of the approximate total mass loss over 68 weeks, we can confidently conclude that VAT reduction is likely to be substantially less on Saxenda than on semaglutide.

Given these considerations, for some people unwilling or unable to take on the risks of bariatric surgery, failing to prescribe semaglutide may then have another effect: it makes these less effective forms of treatment the only viable options available.^[^^iii^^]^ Given the higher cost of semaglutide, it is worth noting that prescribing but failing to subsidise may have the same effect.

All other things being equal, best medical practice suggests that we should not limit persons to more risky or less effective options when better health alternatives are available. Given the efficacy of semaglutide, and its relative risks, this would provide good reason to favour its prescription, and potentially subsidisation, over other forms of intervention when applicable.

Importantly, however, all other things are not equal. Semaglutide *is* more costly than Saxenda, and bariatric surgery, although higher risk, *may* also be cheaper in the long run than both GLP-1s. The out-of-pocket costs of bariatric surgery in the USA, for example, are around $15 000 to $25 000.^46^ Ozempic, on the other hand, as already mentioned, can cost between $3466 and $4333.33 annually. Saxenda can be as low as $2390 annually. As such, after anywhere between 3.5 and 10.5 years, a patient could have spent as much as the cost of bariatric surgery on GLP-1s and would still be required to pay more, given that such medication needs to be taken indefinitely. The exact financial cost of bariatric surgery, however, is unclear. This is because bariatric surgery is also not a permanent weight loss fix, and many healthcare providers recommend weight loss medication in addition to bariatric surgery to manage post-surgery weight gain.^46^ As such, patients may bear the cost of weight loss medication, such as semaglutide and Saxenda, *in addition* to the high cost of the surgery.

What then to make of this complex situation? The best course of action for any given patient will depend on individual circumstances, needs and the actual relative financial burden. What is clear, however, is that given the lower risk profile of semaglutide and the varying needs of patients, semaglutide should be part of that consideration.

### Harm minimisation: compounding, fakes and social inequality

Another reason to prescribe semaglutide for weight loss is that doing so can minimise scarcity-related harms.

The recent surge in popularity of semaglutide has outrun current manufacturing capacities leading to a worldwide shortage of the medication.^47^ As a result, those who rely on semaglutide for diabetes and/or weight loss management have had to turn to alternatives.^48^ In some cases, people have turned to the use of compound medications. Compounding is the practice of combining, mixing or altering ingredients to create medication. In those countries that approved the practice, the purpose is to allow modification of existing pharmaceuticals or the creation of new variations to meet the specific needs of individual patients. In countries like Australia and the USA, however, some pharmacists are compounding and mass-producing semaglutide-like products.^47 49^ This is concerning because compound drugs are not subject to the same regulations for quality, safety or efficacy as other pharmaceuticals. When produced on a mass level, large numbers of patients are exposed to unregulated and potentially harmful pharmaceuticals.^[iv^^]^ This is also especially troubling given reports from the US Food and Drug Administration (FDA) that inspection of *illegal* compounding facilities revealed ‘toaster ovens used for sterilisation, pet beds near sterile compounding areas, and operators handling sterile drug products with exposed skin, which sheds particles and bacteria…’^49^

In addition to these worries, the surge in popularity has also seen the creation of a new market in counterfeit pharmaceuticals: products claiming to be ‘Ozempic’ containing no semaglutide and, in some cases, containing harmful substances associated with miscarriage, loss of consciousness, acute kidney injury, pancreatitis and death^47 49^ Both the FDA and Australia’s Therapeutic Goods Administration (TGA) have issued warnings to the public of using unverified forms of the drug and the TGA is also seeking to tighten regulations on compound pharmaceuticals to prevent the risks associated with mass-manufacturing.

Tighter regulations may deter pharmacies from mass-producing semaglutide. Importantly, history has shown that preventing the manufacturing of substances, especially those that are in high demand, does not solve all the related health and social issues. Doing so can create more or different issues by pushing manufacturing and distribution ‘under-ground’ beyond the reach of regulatory hands. When possible, prescribing semaglutide for weight loss could help to mitigate these harms by deterring people from seeking alternative means.

Another concern with respect to distribution is the widening social inequality that could result from a governmental and healthcare-imposed scarcity or unaffordability. If semaglutide is not made accessible to those of lower income wanting to pursue weight loss, they may feel compelled to take the more ‘affordable’ higher risk options of the mass-compounded or counterfeit varieties or find that the drug is not an option open to them. As Dr. Emma Beckett aptly claims ‘weight-loss drugs aren't a silver bullet. And the conversation around them is still problematic and rooted in diet culture. But they shouldn’t become yet another example of a tool just for rich people’.^50^ When possible, prescribing and potentially subsidising semaglutide for weight loss could then help to ensure that this is not the case.

Novo Nordisk is reportedly spending more than $6 billion to expand manufacturing capacities with the aim of meeting demand.^51^ As such, the global supply issue is set to improve. The current situation, however, *is* one of scarcity. This means that decisions about priority do have to be made. One such decision is how to prioritise treatment for type II diabetes and weight-related health issues. We will say more about this in the section below titled ‘semaglutide shortages for type II diabetes.’

### Promoting autonomy: semaglutide as a motivational enhancer

For some people, losing weight will be a personal goal and may be so quite apart from health. A person, for example, may pursue weight loss for aesthetic reasons, for reasons of physical comfort or as a means to pursue further ends. One final reason for prescribing and subsidising semaglutide for weight loss then is that it can be a helpful tool in achieving these ends, promoting autonomy.

Semaglutide can help persons achieve their goals in similar ways to other motivational enhancers. The term ‘motivational enhancer’ refers to a wide array of pharmaceuticals used to assist an agent’s performance either by enhancing ‘the agent’s will or drive via biomedical means’.^52^ In elite sports, motivational enhancers, like Modafinil, are sometimes used to make engaging in a task or preparation for that task (eg, preparing for a marathon) more enjoyable. Amphetamine-based substances like Ritalin are sometimes used to make performing or preparing a task more bearable by increasing endurance. Like other motivational enhancers, use of semaglutide can make the pursuit of one’s goals more enjoyable and increase endurance in their pursuit.

Indeed, in order to lose weight when taking semaglutide, engaging in regular exercise and a nutrition focused, calorie restricted diet are recommended.^6^ People are often advised for nutritional reasons to eat more slowly.^53^ For those interested in weight loss, the slowing of gastric emptying that semaglutide induces slows digestion and so may help those taking the drug to achieve that goal by making enduring a calorie restricted diet more bearable. An initial loss of weight may also assist in further weight loss as activities like cardiovascular exercise are made easier to engage in either because it becomes more enjoyable or more bearable to perform.

Semaglutide may even be preferable as a weight loss strategy in terms of promoting autonomy. There are many modes of facilitating behaviour change. For example, Antabuse makes alcoholics nauseous if they drink alcohol. Bariatric surgery makes a person feel full and even sick if they eat too much. In many of these cases, the desire to engage in the unhealthy behaviour remains. Indeed, desire can still dominate a person’s mental space, hijacking their attention. Semaglutide is different—it frees a person from the problematic desire. They no longer want to eat at unhealthily high quantities. This frees up their mental space to pursue their goals and to have a better life. In a way, semaglutide enables a person to choose their own desires. This realises freedom and autonomy, on a higher order account of autonomy as desiring to desire.^54^

Insofar, as semaglutide is a motivational enhancer, and motivational enhancers are tools that assist users in achieving ends that are valuable to them, this would seem to provide a strong prima facie reason to also endorse the prescription of semaglutide independently of any therapeutic motivation.

## Four objections to the use of semaglutide for weight loss

### Semaglutide shortages for type II diabetes

One of the most important and pressing objections against prescribing semaglutide for weight loss is that it will lead to a shortage of the drug for those who ‘need it most’—those with type II diabetes. Activist and director of Diabetes Cymru Rachel Burr, for example, claims that ‘[People are] sitting at home, they’re reading the news, and it can be a very stressful thing to be thinking that their medication may run out…we would certainly like to see clinicians adhering to the guidance that’s been given. It should only be prescribed for people…living with type 2 diabetes’.^55^

People may request semaglutide for a range of purposes, including aesthetics, to treat conditions that are less urgent than treating type II diabetes or to enhance their capacity to pursue other ends that they care about. Burr is certainly correct that priority must be given to those who need it most when prescribing semaglutide. What is not clear, however, is that this necessitates *only* prescribing semaglutide to treat type II diabetes. Given the potential health benefits of losing weight for persons with high levels of visceral fat, poor cardiovascular health and other health-related issues, prescribing semaglutide in these cases may address greater or more pressing health concerns than treating type II diabetes with semaglutide—where type II diabetes may still be treated by other medications such as metformin. So, if priority must be given to those in most need, it is evident that people with weight-related health issue should not be excluded from this assessment. Moreover, they should also, under the right conditions, even be given priority access to semaglutide when need demands.

### Semaglutide is the ‘easy way out’ for individuals

Another common objection against the use of semaglutide for weight loss is that it is ‘the easy way out’ for those who want to lose weight. As described earlier, Rosenberg describes it as ‘common wisdom’ that using Wegovy is cheating. Similar criticisms have been launched against elite athletes using motivational enhancers. According to some bioconservative views, taking motivational enhancers falls into the category of ‘no pain, no praise’.^52^ Like weight loss drugs, motivational enhancers are considered to be an athlete’s ‘easy way out.

Those who object to the bioconservative view, however, rightly argue that motivational enhancers are far from the ‘easy way out’.^52 56^ This is because in order to be successful, athletes cannot simply rely on doping to achieve their ends. Success often still requires gruelling effort and dedication on the athlete’s part. Athletes, for example, still need to follow a rigorous and often high-intensity training regime, control diet and forgo other meaningful projects in the pursuit of their ends. Moreover, it also requires taking on significant risks in pursuit of their goals. Side effects from amphetamines, for example, include irregular heartbeat, increased blood pressure, memory loss and increased risk of stroke.^57^ Athletes who take motivational enhancers are also at a higher risk of injury due to the toll of increased exertion the enhancers allow for. It is for this reason that defenders of motivational enhancers claim that these drugs should not be viewed as a ‘cheat’s way out’ but as a tool in the pursuit of a worthy end compatible with praise.

Like the use of motivational enhancers in sports, it is evident that semaglutide does not make effort redundant. Weight loss success on semaglutide requires a prolonged resolute dedication to medical intervention because semaglutide is only beneficial for weight loss for as long as people continue to take it.^38^ Moreover, as numerous studies emphasise, weight loss is often most successful when medication is taken in combination with exercise and a calorie-restricted, nutrition focused diet—diets that often require special preparation and result in energy deficits.^6^ In addition, success when taking semaglutide also requires taking on significant risks with respect to known and potential side effects. Those who take semaglutide also, and often knowingly, place themselves in environments that they have and will risk again being discriminated against in pursuit of their ends. Medical institutions are well-recognised as often being hostile and discriminatory places for fat people.^58 59^ This discrimination can contribute in deep ways to damage self-esteem and can also result in real medical negligence and loss. Here then, the risk is not seeking out semaglutide per se but placing oneself in a known hostile environment in order to seek treatment.

Given the real effort and risks people take on with semaglutide, like those who take motivational enhancers in elite sports, it is clearly not the ‘easy way out’. Nonetheless, many patients still choose to take semaglutide. This is presumably—and as expressed in the personal accounts described earlier—because they deem the ends that they are aiming for as worth the effort and risk and see semaglutide as an effective tool to assist them in achieving those ends.

### Semaglutide is the ‘easy way out’ for governments

Some commentators are concerned about the availability of semaglutide and its potential impact on government policy. In particular, they are concerned that governments will view semaglutide as an ‘easy way out’ with respect to dealing with population obesity when it is a healthcare problem, rather than tackling what they see as the ‘root causes’. Professor Giles Yeo, as mentioned earlier, is one such commentator. Journalist Sarah Boseley is another. She writes ‘Governments want a quick fix, and these drugs seem to offer one’.^60^

Both commentators claim that if governments are to take seriously the health of their populations, they will need to support policy that promotes nutrition-focused eating. This is surely correct. It is also possible that governments may divert resources from efforts to address deeper structural problems associated with obesity-related health issues, such as financially inaccessible healthy food options and health-promoting work arrangements that provide leisure time for physical exercise. Currently, governments have not opted to prioritise semaglutide, as evidenced by how few offer subsidisation for the medication. That may change, especially if the cost of semaglutide becomes more affordable. This risk, however, does not provide reason *not* to prescribe semaglutide for weight loss, but it does provide reason to monitor governments and hold them accountable for addressing the deeper structural issues.

### Semaglutide use reinforces harmful fat-phobic rhetoric

One final and important criticism made against the use of semaglutide for weight loss is that widespread use will harm fat people and contribute to and reinforce fat-phobia.

Weight loss within the global context is intimately tied up with conceptions of virtue and moralisation. Fatness, as Oswald describes, ‘is discursively constructed as immoral [and] fat loss (that is, bringing the body back into alignments with oppressive bodily norms) is considered a moral activity’’—provided that it is done the ‘right way’.^4^. Those who are fat are often cast as lazy and lacking the virtues of will-power and wisdom (‘if only you exercised more, had better self-control, made better choices’). Those who are slim, or lose weight are, conversely, often celebrated.^[^^v^^]^ It is indeed for this reason that the use of semaglutide for weight loss is often cast as cheating or ‘the easy way out’; it supposedly circumvents the virtues of effort and will-power required for (but by no means ensured) weight loss, and so any achievements are considered ill-gained.

The moralisation of fatness is worrisome because it has real-world consequences for the physical and mental health of fat people, as well as the wider life opportunities made available to them. A 2018 study examining the research done on the relationship between weight stigma—defined as ‘the social rejection and devaluation that accrues to those who do not comply with prevailing social norms of adequate body weight and shape’—and health, for example, found that weight stigma ‘is harmful to health, over chronic diseases and conditions’.^59^ Tomiyama *et al.* explain that weight stigma ‘can trigger physiological and behavioural changes linked to poor metabolic health and increased weight gain’ including decreases in self-regulation, increased cortisol levels and avoidance of physical activity.^59^ In addition, Tomiyama *et al.* explain that persons ‘who perceive that they have been discriminated against on the basis of weight are roughly 2.5 times as likely to experience mood or anxiety disorders as those that do not, accounting for standard risk factors for mental illness and objective BMI’.^59^ In terms of health-related opportunities, fat patients were found to be regarded with less respect (described by physicians as ‘lazy’, ‘weak-willed’, ‘bad’ and a ‘waste of time’) by healthcare providers and offered less comprehensive care from those providers than persons with a ‘healthy’ BMIs. As a result, people with high BMIs reported reluctance to seek out treatment from fear of discrimination. In addition to poorer health and healthcare opportunities, it also is widely recognised that weight stigma and the associated harms extend to many other areas of life including employment opportunities, educational opportunities and public accommodations.^61^

The concern regarding the widespread use of semaglutide for weight loss with respect to fat-phobia is multifaceted. One concern is that healthcare providers will over prescribe semaglutide for weight loss without investigating the pressing healthcare needs of the patient. As O’Neil explains ‘people with larger bodies often report that when they go to the doctor, their problems are ignored or written off as an inevitable result of their weight. Without asking questions, they say, healthcare providers suggest diets they’ve already tried, and lifestyle changes they’ve already made’.^62^ The concern with semaglutide and the hype surrounding it here then is that it will exacerbate such bad practice.

Another concern is that the widespread use of semaglutide will reinforce and exacerbate the already harmful fat-phobia and the moralisation of being fat by, for example, further invalidating ‘fat existence’ (eg, there is ‘no excuse’ anymore), by cementing the idea that being overweight is an individual choice (eg, there is a ‘cure’, and you’re ‘choosing’ not to take it) or cementing overlapping oppressive ideologies (eg, ‘if you cannot take it because you cannot afford it, you need to work harder’—here being ‘lazy’ is framed both as a cause of fatness, and its maintenance, thus intersecting with class ideology). With respect to the last point, Osward claims that ‘the belief that people are obligated to work hard to lose fat may transition slightly to a belief that those who remain fat are obligated to work hard to earn money in order to access fat loss—an oppressive and impossible condition under oppressive economic structures’.^4^

The concerns cited here with respect to fat-phobia and weight-related stigma are certainly warranted. What they demonstrate, however, is again not that semaglutide ought not be prescribed or subsidised—and, in fact, in some cases, these arguments favour subsidisation as a matter of equal access and opportunity. What they do demonstrate is a pressing need to address weight-related stigma and misconceptions as a matter of individual and collective health in its own right. Continuing to celebrate semaglutide as a ‘miracle drug that will end the obesity epidemic’ will not do this. Nor, however, will denying people access to a drug that is potentially lifesaving. Moreover, it may be the case that even if widely proscribing semaglutide does contribute to fat-phobia, at the least or especially for those persons who need it in potentially lifesaving ways, the social health benefits may outweigh the harms. In parallel to prescribing semaglutide then, there should be concerted public and professional education on the causes of obesity, healthy lifestyle choices and respect for patients in order to address misconception and discrimination.

## Semaglutide for weight loss: a drug for all?

Many countries have already deemed semaglutide sufficiently safe and permissible for use in adults, and while in many cases decisions have been made about who has access to semaglutide, it is important to reiterate that there is still much that is unknown. Many studies that analyse semaglutide’s efficacy, for example, do so by focusing primarily on reductions in BMI, which are inaccurate indicators of health.^63^ Population samples in the studies that have been conducted also typically exclude persons with pre-existing conditions, as well as those that fall into other vulnerable risk categories, making the results unknown for people who fall into those categories. These groups include persons with a history of myocardial infarction, stroke or cardiovascular complications, as well as breastfeeding persons, persons assigned female at birth of childbearing age and persons with a history of suicide attempts.^64^ In addition, the data are also limited because the outcomes of long-term usage remain unknown. When data are unknown—and particularly in multiple, compounding ways—the risks of using semaglutide are higher.

Many of the study limitations and unknowns noted here are not unique to studies of semaglutide. Studies of the efficacy of bariatric surgery also employ BMI as a measure and, as mentioned earlier, also lack long-term data on side effects and efficacy. Nonetheless, it is considered a gold standard treatment. Many other studies also exclude vulnerable or otherwise high-risk participants from their studies.^65^ These considerations then are not decisive reasons against semaglutide prescription. They are, however, reason to proceed with caution when prescribing, especially for populations excluded from existing studies.

There are also, as we have noted, very promising results for health outcomes that should not be overlooked, including improved liver and cardiovascular functioning, as well as lower rates of stroke, kidney and heart disease, in addition to the benefits for the treatment of type II diabetes. Among other things, semaglutide can also provide a safer treatment option relative to other, more aggressive therapies; its prescription can minimise government-mandated scarcity-related harms, and it can be an effective tool to promote and respect autonomy.

Given the limited knowledge we have, as well as the known and potential risks and benefits of semaglutide use, how should we proceed? It is our view that when production capacity demands are met, using semaglutide should be an option generally made available to patients for weight loss within the limits of responsible ethical practice.^[vi]^ Should the medication prove to be as or more cost-effective than existing publicly funded options, such as bariatric surgery, it is our view that this would also provide reason for government subsidy. Given that scarcity *is* a pressing issue, it is also our view that priority access to the drug be given to those most in need, where this includes persons seeking the drug for type II diabetes *and* weight loss health–related issues when the conditions of need are met.

## Data Availability

No data are available.

## Footnotes

[i] Rybelsus is also used to treat type 2 diabetes, and it is the first oral GLP-1 that has been approved by the US Food and Drug Administration (FDA) for the treatment of type II diabetes. Rybelsus tablets contain 7 or 14 mg of semaglutide, and it is taken daily.

[ii]With respect to semaglutide, it is also worth noting that there is a history of medications repurposed for weight loss, such as Phen-Fen, ending in disaster due to unforeseen long-term harms.

[iii]For many, not even this option will be obtainable due to economic constraints.

[iv]This is not to claim that compounding itself is untrustworthy; it is an integral part of healthcare when used for the purpose of tailoring medications to meet individual needs. It also has the potential to meet demand shortages if regulated properly. As it stands, however, these regulations are not in place, and so if compounds are mass manufactured, they presented the risk described.

[v]Though, of course, sometimes persons are also criticised for being ‘too thin’.

[vi]Prescribing to patients with insufficiently high BMI, for example, or a history of eating disorder would constitute unethical practise.
